# The Effectiveness of a Smartphone Intervention Targeting Suicidal Ideation in Young Adults: Randomized Controlled Trial Examining the Influence of Loneliness

**DOI:** 10.2196/44862

**Published:** 2023-03-30

**Authors:** Lauren McGillivray, Nicholas Keng-Meng Hui, Quincy J J Wong, Jin Han, Jiahui Qian, Michelle Torok

**Affiliations:** 1 Black Dog Institute University of New South Wales Sydney Australia; 2 Faculty of Medicine and Health University of New South Wales Sydney Australia; 3 School of Psychology Western Sydney University Sydney Australia

**Keywords:** loneliness, suicidal ideation, suicide prevention, digital therapeutics, smartphone intervention, apps

## Abstract

**Background:**

Loneliness is commonly reported by young people and has been shown to contribute to the rapid onset and escalation of depression and suicidal ideation during adolescence. Lonely people may also be particularly susceptible to disengaging from treatment early given the likelihood of their more complex clinical profiles leading to cognitive fatigue. While a smartphone intervention (LifeBuoy) has been shown to effectively reduce suicidal ideation in young adults, poor engagement is a well-documented issue for this therapeutic modality and has been shown to result in poorer treatment outcomes.

**Objective:**

This study aims to determine whether loneliness affects how young people experiencing suicidal ideation engage with and benefit from a therapeutic smartphone intervention (LifeBuoy).

**Methods:**

A total of 455 community-based Australian young adults (aged 18-25 years) experiencing recent suicidal ideation were randomized to use a dialectical behavioral therapy–based smartphone intervention (LifeBuoy) or an attention-matched control app (LifeBuoy-C) for 6 weeks. Participants completed measures of suicidal ideation, depression, anxiety, and loneliness at baseline (T0), post intervention (T1), and 3 months post intervention (T2). Piecewise linear mixed models were used to examine whether loneliness levels moderated the effect of LifeBuoy and LifeBuoy-C on suicidal ideation and depression across time (T0 to T1; T1 to T2). This statistical method was then used to examine whether app engagement (number of modules completed) influenced the relationship between baseline loneliness and suicidal ideation and depression across time.

**Results:**

Loneliness was positively associated with higher levels of overall suicidal ideation (*B*=0.75, 95% CI 0.08-1.42; *P*=.03) and depression (*B*=0.88, 95% CI 0.45-1.32; *P*<.001), regardless of time point or allocated condition. However, loneliness did not affect suicidal ideation scores across time (time 1: *B*=1.10, 95% CI –0.25 to 2.46; *P*=.11; time 2: *B*=0.43, 95% CI –1.25 to 2.12; *P*=.61) and depression scores across time (time 1: *B*=0.00, 95% CI –0.67 to 0.66; *P*=.99; time 2: *B*=0.41, 95% CI –0.37 to 1.18; *P*=.30) in either condition. Similarly, engagement with the LifeBuoy app was not found to moderate the impact of loneliness on suicidal ideation (*B*=0.00, 95% CI –0.17 to 0.18; *P*=.98) or depression (*B*=–0.08, 95% CI –0.19 to 0.03; *P*=.14).

**Conclusions:**

Loneliness was not found to affect young adults’ engagement with a smartphone intervention (LifeBuoy) nor any clinical benefits derived from the intervention. LifeBuoy, in its current form, can effectively engage and treat individuals regardless of how lonely they may be.

**Trial Registration:**

Australian New Zealand Clinical Trials Registry ACTRN12619001671156; https://tinyurl.com/yvpvn5n8

**International Registered Report Identifier (IRRID):**

RR2-10.2196/23655

## Introduction

Suicidal ideation is a serious condition affecting approximately 11% of all young people [1,2] and has been shown to increase the risk of suicide attempt and suicide death approximately 2-fold [2-4]. Understanding which young people have an elevated risk of ideation is critical to improving early detection and shaping early intervention initiatives that can effectively prevent premature mortality in young people. There are many reasons why young people may experience suicidal ideation, including mood and affective disorders [5], trauma [6], and relationship issues [7], among other factors. One condition that has not received much attention in understanding suicidal trajectories is loneliness, which is a common manifestation of and highly comorbid with complex mental health and trauma experiences [5,6,8,9].

Loneliness has been defined as the unpleasant subjective experience of feeling isolated when a discrepancy exists between the actual and the desired levels of interpersonal relationships [10]. Research has found that loneliness is more prevalent in adolescents and young adults than in working-aged adults [11] and that it can contribute to the rapid onset and escalation of depression [8,9] and suicidal ideation [11,12] during adolescence. Findings from The Young Australian Loneliness Survey [13] found that one-quarter of young people surveyed (ages 12-25 years) reported clinically significant levels of loneliness, and young adults in particular (ages 18-25 years) were lonelier than adolescents (ages 12-17 years). These findings suggest that loneliness may be developmentally important to the onset and escalation of suicidal ideation in young people. Accordingly, further confirmatory research to establish the impacts of loneliness on young people’s mental health is warranted.

Recent research examining the social and health impacts of the COVID-19 pandemic suggests there is a growing epidemic of loneliness among young people [14]. Many public health measures implemented to contain the pandemic enforced physical isolation from friends and family, and may have led to disproportionate negative mental health effects for young people [15-17], as social connections with peers play a vital role in their healthy development [18]. For example, there is evidence of worsening rates of depression [19] and marked increases in hospital presentations for suicidal ideation and self-harm [20] among young people since the onset of the pandemic. There are, however, major gaps in our understanding of how loneliness may be contributing to these trends, particularly how it may have impacted help-seeking behaviors for mental health issues [21]. Though limited, there is some evidence that more severe loneliness is associated with greater external or professional help-seeking, driven by a diminished perception of being able to self-manage mental health symptoms [21,22]. Related to these knowledge gaps on links between loneliness and help-seeking, there is a dearth of research examining how loneliness may affect how young people interact with, and benefit from, help they do access given potential comorbidities with complex psychopathology [10,11]. Specifically, there is no research yet on this issue in relation to digital interventions for mental health, which increased in availability during the pandemic when face-to-face services were limited [23].

Meta-analytic research on the effectiveness of self-guided digital health interventions for suicide prevention shows that these digital tools can help to reduce the severity of suicidal ideation [24-27], and there is also evidence that the level of engagement with digital health interventions is a significant positive moderator of clinical benefit [28,29]. However, issues of poor engagement with digital health interventions are well documented, with an average of 70% of users not completing all treatment modules [28], yet reasons for poor engagement are not fully understood. The few research studies that have investigated predictors of engagement have focused on the intervention itself (eg, desirable content or interface [30-32]) rather than on user characteristics. Attrition data from clinical trials have noted higher dropout rates among individuals with more clinically severe profiles (eg, severe depression) due to the psychological effort required to attain treatment goals [29,33]. Given that loneliness is a significant and independent risk factor for complex psychopathology, including depression and suicidal ideation [8,9,11,12,34-36], lonely people may be particularly susceptible to disengaging from treatment early. Establishing what role loneliness plays in how young people engage with and subsequently benefit from digital health interventions will help to advance current understandings of whether specific strategies are needed to support adherence, which is particularly relevant to ensuring supports are being accessed optimally during periods of heightened loneliness and mental ill health, such as health epidemics or pandemics.

This study presents a secondary and post hoc exploratory analysis of data from a randomized controlled trial that tested the efficacy of a therapeutic smartphone intervention (LifeBuoy) against an attention-matched placebo control app in supporting young adults to self-manage suicidal ideation. The trial took place during the COVID-19 pandemic. The main analyses showed that LifeBuoy was associated with significant reductions in the severity of suicidal ideation in the intervention condition compared to the control group [25]; however, a better understanding of the fundamental person-specific characteristics that may influence outcomes will help inform who self-guided digital health interventions should be targeted to:

- Aim 1: Determine whether baseline loneliness severity moderates change in suicidal ideation and depression scores across time among young adults in both the intervention and control conditions
- Aim 2: Examine whether engagement with LifeBuoy (defined as the number of modules completed) moderates the impact of loneliness on suicidal ideation and depression in the treatment (exposure) condition

## Methods

This paper is a secondary analysis of a clinical trial of the LifeBuoy smartphone intervention, with the full trial details, safety protocols [37], and main outcomes reported elsewhere [25]. The trial protocol was prospectively registered on the Australian New Zealand Clinical Trials Registry (ACTRN12619001671156) and has also been published elsewhere [37]; the LifeBuoy intervention was registered with the Therapeutic Goods Administration Clinical Trial Notification scheme (CT-2020-CTN-00256-1-v1).

### Ethics Approval

Ethics approval for this study was obtained from the University of New South Wales Human Research Ethics Committee (HC190764). All procedures performed in the study involving human participants were in accordance with the ethical standards of the institutional or national research committee and with the 1964 Helsinki Declaration and its later amendments or comparable ethical standards. As such, informed consent to participate was obtained from all participants prior to data collection.

### Participants and Recruitment

The study was a 2-arm parallel, double-blind, randomized controlled trial conducted at the Black Dog Institute (BDI), Australia. Participants were recruited from the community via targeted advertisements posted on Facebook between May 11 and May 22, 2020 (Multimedia Appendix 1). Eligibility criteria were as follows: aged between 18 and 25 years, fluent in English, own and have access to a smartphone, currently residing in Australia, and having experienced suicidal thoughts in the past 12 months. Participants were excluded if they reported being diagnosed with a psychotic disorder ever or had attempted suicide in the 1 month prior to trial registration due to safety considerations. Participants undergoing other mental health treatments were not excluded from the trial. The study was approved by the University of New South Wales Human Research Ethics Committee (HC190764) and required written opt-in consent from participants.

### Procedure

After completing the baseline survey, participants were assigned randomly (2:2) to the intervention or control condition within a block design (four per block). Randomization was performed using a computer-generated algorithm integrated into BDI’s digital trial management platform. All study participants and investigators were masked from condition assignment until the completion of the final survey.

The trial was run online using this trial management platform, which also supported the recruitment portal, including online consent, screening, registration, data collection, and access to the apps (see [25] for full details). Participants were required to complete these closed (password-protected) surveys within 7 days at baseline (T0, day 0), post intervention (T1, day 43), and 3 months post intervention (T2, day 132). After completing the baseline assessment, participants were sent a link (via email and SMS text message) to download the free app randomly allocated to them and informed that they had 6 weeks to access this app. No reminders/prompts or support were provided to use the apps.

### Intervention

LifeBuoy is a brief self-guided smartphone intervention consisting of seven models grounded in dialectical behavior therapy and acceptance and commitment therapy. Each module takes approximately 5 minutes to complete, and participants had the opportunity to return to them as frequently as needed over the course of 6 weeks. Participants were required to complete each module sequentially before moving on to the next. Each module introduced and practiced knowledge and skills relating to distress tolerance, emotional regulation, and goal setting through interactive learning exercises. A detailed description of LifeBuoy has been published elsewhere along with screenshots of the app [25].

### Control

A matched-attention placebo app (LifeBuoy-C) was developed for the control condition (details published elsewhere) [25]. LifeBuoy-C provided generalized mental health information in a similar format and time expectancy to the intervention app to control for potential digital placebo effects [38]. LifeBuoy-C mirrored the module structure, user interface, and graphic design of LifeBuoy but contained no therapeutic content.

### Measures

#### Suicidal Ideation (Primary Outcome)

Suicidal ideation was measured using the Suicidal Ideation Attributes Scale (SIDAS) [39]. The SIDAS comprises five items rated on an 11-point scale that assess the frequency, severity, impact, and controllability of suicidal ideation. Total scores range from 0 to 50, with higher scores signifying more severe suicidal ideation. Scores ≥21 indicate a high risk for suicidal behavior. The scale has demonstrated acceptable internal consistency (Cronbach α=.91) [39].

#### Loneliness

Loneliness was measured using the Three-Item Loneliness Scale (TILS) [40], which assesses social exclusion, lack of companionship, and social isolation. The TILS is based on the University of California, Los Angeles Loneliness Scale [40] and has demonstrated good internal consistency (Cronbach α=.72) [40]. Items are rated on a 3-point Likert scale from hardly ever (1) to often (3), with a higher combined score (ranging from 3 to 9) indicating greater loneliness.

#### Depression

Depression was measured using the Patient Health Questionnaire (PHQ-9). The PHQ-9 assesses the nine diagnostic criteria of depression from the *Diagnostic and Statistical Manual of Mental Disorders* (Fourth Edition) [41]. Respondents rate their responses on a 4-point Likert scale that ranges from not at all (0) to nearly every day (3). Total scores range from 0 to 27, with higher scores indicating greater depression severity. The scale has demonstrated good internal consistency (α>.80) [42].

#### App Engagement

The LifeBuoy app recorded details about the number of modules completed and automatically uploaded this data to central servers. Module completion was recorded at the time of baseline (T0) and at the postintervention (T1) and 3-month postintervention follow-up (T2).

### Statistical Analysis

To determine whether the loneliness levels of participants at baseline moderated the effect of the LifeBuoy and LifeBuoy-C apps on suicidal ideation (SIDAS) and depressive symptoms (PHQ-9) across time (T0 to T1; T1 to T2), piecewise linear mixed models for repeated measures analyses were used. Thus, loneliness (TILS), two time variables reflecting T0 to T1 and T1 to T2, condition (LifeBuoy, LifeBuoy-C), and relevant interaction terms between these variables were specified as fixed effects in models, along with an intercept as a random effect. An identity covariance matrix was used, and degrees of freedom were estimated using Satterthwaite’s method. The linear mixed model approach can include all participants in the analyses, even those with missing follow-up data points, under the missing-at-random assumption, and therefore accords with the intention-to-treat principle.

To examine within the LifeBuoy condition whether app engagement affected the relationship between loneliness and each of the key outcomes (suicidal ideation, depression), piecewise linear mixed models for repeated measures analyses were conducted similar to the above approach described. In this case, app engagement (the number of modules completed), loneliness (TILS), two time variables reflecting T0 to T1 and T1 to T2, and relevant interaction terms between these variables were specified as fixed effects in models, along with an intercept as a random effect. Broader engagement data (number of modules completed by condition and median time spent in the app by condition) has been reported elsewhere [25]. Safety data and harms have also been reported elsewhere [24].

For all analyses, *P* values ≤.05 were considered significant. For all linear mixed models examined, residual plots indicated no distinct patterns, and the distribution of residuals in histograms approximated normality, indicating model assumptions were met. SPSS 25.0 (IBM Corp) was used for all analyses. Attrition analyses were conducted and reported elsewhere [25].

## Results

### User Statistics

In total, 455 young adults completed the baseline survey and were randomized to either the intervention condition (n=228) or the control condition (n=227). No participants formally withdrew or were excluded from the study. Demographic and clinical profiles of the total sample are described elsewhere, along with a CONSORT (Consolidated Standards for Reporting Trials) trial flow diagram [25]. The mean loneliness score at baseline was 7.17 (SD 1.83), with no significant differences between the intervention and control conditions (7.19 vs 7.16; *F*=0.89; t_453_=0.16; *P*=.44). The mean SIDAS score was 22.61 (SD 8.18), which indicates clinically relevant levels of suicidal ideation (scores ≥21), with 136 (59.6%) participants in the intervention condition and 129 (57.1%) participants in the control condition meeting this criterion. The mean PHQ-9 score was 17.15 (SD 5.64), indicating that the sample, on average, met the clinical threshold for moderate-severe depression (scores ≥15). A total of 152 (66.7%) participants in the intervention condition and 156 (69.3%) participants in the control condition met the moderate-severe depression threshold. There were 202 (90.2%) participants in the intervention condition and 199 (88.4%) participants in the control condition who endorsed ever receiving mental health treatment. In the intervention condition, there was no significant difference in the number of modules completed between those who have (mean 6.80, SD 4.41) and have not (mean 6.77, SD 2.84) ever received mental health treatment (*F*_1,222_<1; *P*=.98). In the control condition, there was no significant difference in the number of modules completed between those who have (mean 6.84, SD 4.96) and have not (mean 5.81, SD 3.82) ever received mental health treatment (*F*_1,223_=1.04; *P*=.31).

In the intervention condition, there was a significant positive correlation between baseline loneliness and PHQ-9 scores (*r*=0.32; *P*<.001) but not between loneliness and SIDAS scores (*r*=–0.03; *P*=.67). In the control condition, significant positive correlations were observed between baseline loneliness and SIDAS scores (*r*=0.16; *P*=.02) and loneliness and PHQ-9 scores (*r*=0.27; *P*<.001).

### Loneliness at T0 as a Moderator of the Effect of LifeBuoy and LifeBuoy-C on Suicidal Thinking and Depression

For the model with suicidal thinking (SIDAS) as the dependent variable (Table 1), there was a significant main effect of loneliness (*P*=.03), indicating that higher levels of loneliness were positively associated with higher levels of suicidal thinking overall, irrespective of condition or time point. There was also a significant time 1 × condition interaction (*P*=.02), as expected given the primary outcomes analyses previously reported [25] reflecting a nonsignificant change in suicidal thinking in the LifeBuoy-C condition (*B*=–3.23, 95% CI –10.48 to 4.01; *z*=–0.87; *P*=.38; *d*=–0.38) but a significant decrease in suicidal thinking in the LifeBuoy condition (*B*=–7.84, 95% CI –14.76 to –0.92; *z*=–2.22; *P*=.03; *d*=–1.00), resulting in a between condition effect size (*d*=0.42) at T1. There were no other significant effects. For the model with depression (PHQ-9) as the dependent variable (Table 1), there was a significant main effect of loneliness (*P*<.001), indicating that higher levels of loneliness were positively associated with higher levels of depression overall, irrespective of condition or time point. There were no other significant effects.

**Table 1:** Loneliness as a moderator of the effect of LifeBuoy and LifeBuoy-C on the SIDAS and PHQ-9.

	DV^a^: SIDAS^b^			DV: PHQ-9^c^	
	*B* (95% CI)	*P* value	*B* (95% CI)		*P* value
Intercept	16.98 (12.03 to 21.94)	<.001	10.86 (7.66 to 14.06)		<.001
Time 1^d^	–0.75 (–8.01 to 6.51)	.84	–2.01 (–5.62 to 1.59)		.27
Time 2^e^	1.15 (–8.01 to 10.32)	.80	2.19 (–1.90 to 6.27)		.29
Condition	6.71 (–0.05 to 13.48)	.05	–0.53 (–4.88 to 3.82)		.81
Loneliness	0.75 (0.08 to 1.42)	*.03^f^*	0.88 (0.45 to 1.32)		*<.001*
Time 1 × condition	–12.52 (–22.56 to –2.49)	*.02*	–0.92 (–5.87 to 4.02)		.71
Time 2 × condition	–2.35 (–14.96 to 10.26)	.71	–1.57 (–7.30 to 4.15)		.59
Time 1 × loneliness	–0.35 (–1.33 to 0.63)	.49	–0.14 (–0.62 to 0.34)		.57
Time 2 × loneliness	–0.36 (–1.59 to 0.87)	.57	–0.32 (–0.87 to 0.23)		.25
Condition × loneliness	–0.87 (–1.78 to 0.05)	.06	0.06 (–0.53 to 0.64)		.85
Time 1 × condition × loneliness	1.10 (–0.25 to 2.46)	.11	0.00 (–0.67 to 0.66)		.99
Time 2 × condition × loneliness	0.43 (–1.25 to 2.12)	.61	0.41 (–0.37 to 1.18)		.30

^a^DV: dependent variable.

^b^SIDAS: Suicidal Ideation Attributes Scale.

^c^PHQ-9: Patient Health Questionnaire.

^d^Time 1: postintervention.

^e^Time 2: 3-month follow-up.

^f^Italicized *P* values denote statistical significance.

### App Engagement as a Moderator of Loneliness’ Relationship With Suicidal Thinking and Depression in the LifeBuoy Condition

Among the LifeBuoy condition (n=228), the mean number of app modules completed was 6.84 (SD 4.27), and loneliness scores at baseline were not significantly correlated with module completion (*r*=0.05; *P*=.42). In examining whether loneliness moderated the relationship between the number of LifeBuoy modules completed and clinical benefits, there were no significant effects in the model with suicidal thinking (SIDAS) as the dependent variable (Table 2). For the model with depression (PHQ-9) as the dependent variable (Table 2), there was only a significant main effect of loneliness (*P*<.001), indicating that higher levels of loneliness were positively associated with higher levels of depression overall, irrespective of time point or engagement.

**Table 2:** App engagement as a moderator of loneliness’ relationship with the SIDAS and PHQ-9 in the LifeBuoy condition.

	DV^a^: SIDAS^b^			DV: PHQ-9^c^	
	*B* (95% CI)	*P* value	*B* (95% CI)		*P* value
Intercept	23.50 (13.48 to 33.52)	<.001	6.44 (0.34 to 12.54)		.04
Time 1^d^	–13.81 (–30.82 to 3.20)	.11	0.56 (–6.97 to 8.08)		.88
Time 2^e^	–10.00 (–32.45 to 12.45)	.38	1.76 (–7.18 to 10.70)		.70
Loneliness	–0.13 (–1.46 to 1.19)	.84	1.47 (0.66 to 2.27)		<.001
Modules	0.03 (–1.32 to 1.39)	.96	0.60 (–0.23 to 1.42)		.16
Time 1 x loneliness	0.83 (–1.37 to 3.02)	.46	–0.53 (–1.51 to 0.45)		.29
Time 2 x loneliness	1.02 (–1.88 to 3.92)	.49	–0.14 (–1.32 to 1.03)		.81
Time 1 x modules	0.08 (–2.17 to 2.33)	.94	–0.54 (–1.56 to 0.48)		.30
Time 2 x modules	1.22 (–1.70 to 4.14)	.41	–0.17 (–1.38 to 1.03)		.78
Loneliness x modules	0.00 (–0.17 to 0.18)	.99	–0.08 (–0.19 to 0.03)		.14
Time 1 x loneliness x modules	–0.01 (–0.30 to 0.28)	.94	0.06 (–0.07 to 0.19)		.38
Time 2 x loneliness x modules	–0.13 (–0.51 to 0.25)	.50	0.03 (–0.12 to 0.19)		.68

^a^DV: dependent variable.

^b^SIDAS: Suicidal Ideation Attributes Scale.

^c^PHQ-9: Patient Health Questionnaire.

^d^Time 1: postintervention.

^e^Time 2: 3-month follow-up.

## Discussion

### Principal Findings

This study investigated whether loneliness played a role in moderating the efficacy of and engagement with a therapeutic smartphone intervention designed to help young people manage suicidal ideation. Baseline loneliness scores were not found to significantly moderate changes in suicidal ideation or depression severity post intervention or at the 3-month follow-up in either condition. The severity or extent of loneliness at baseline also had no effect on how young adults self-engaged with the LifeBuoy app, contrary to prior research that suggests that lonelier individuals may lack self-efficacy to manage mental health issues [21,22].

These results are consistent with the existing literature examining the relationship between loneliness and mental health to the extent that they suggest that loneliness is associated with more complex psychopathology [8,9,11,12,34-36]. In this study, this relationship was evidenced by significant positive correlations between loneliness and suicidal ideation and depression scores. However, these findings go beyond confirming simple relationships between loneliness and mental health. This study shows that despite loneliness being associated with poorer mental health, it does not appear to be a characteristic or condition that moderates how young people engage with, nor benefit from, a digital health intervention. Though prior studies have posited that positive associations between depression and treatment dropout may be moderated by the psychological effort required to sustain treatment adherence [31,43], this study suggests that loneliness is not an independent nor significant factor that exacerbates this nonadherence, at least for brief smartphone interventions.

One potential explanation for the null association is that there was a high prevalence of moderate-severe levels of suicidal ideation and depression across the total sample, which may have obscured or dampened the effects of transdiagnostic conditions such as loneliness. Alternatively, the proportion of participants reporting clinically severe suicidal ideation (SIDAS score ≥21) may be important to consider in efforts to understand why loneliness did not emerge as a unique predictor of engagement nor benefit in this study. The LifeBuoy app was purposefully designed to improve ideation [25], and the trial was marketed to young people for whom ideation was currently or recently a salient concern. As such, LifeBuoy may have been seen as an intervention highly relevant to the needs of the young people who participated in the trial—and need is a strong intrinsic motivator for engagement [44]. Indeed, young people in both conditions engaged similarly well with the smartphone apps over the course of the trial [25]. Actual high levels of motivation or need to engage with the app may have overridden any cognitive barriers typically associated with loneliness. Examining the role of loneliness in a more clinically heterogenous or diverse sample of young adults may help to clarify the relationships between mental ill health, loneliness, and engagement with treatment. As this is the first study to examine the role of loneliness in how young people experiencing suicidal ideation benefit from a digital health intervention, caution must be taken against concluding that loneliness is not an important factor in understanding engagement with, and benefit from, self-guided treatment.

Overall, our findings show that participants engaged with and benefited from LifeBuoy, irrespective of loneliness severity, which shows that LifeBuoy, in its current form, can effectively engage and treat individuals despite how lonely they may be. This finding strengthens the potential for digital health interventions to become an established mental health treatment method for all young people experiencing suicidal ideation. These findings also suggest that a digital therapeutic such as LifeBuoy might be suitable for large-scale delivery during future pandemics or environmental crises when loneliness is expected to increase.

The study findings also add new knowledge about which person-specific characteristics or conditions may be important to target using digital health interventions to enhance adherence and clinical benefits. Loneliness, as an isolated factor, may not be important to uniquely target future digital health intervention development when samples or users present with complex psychopathology.

Although this study found no significant link between baseline loneliness and depression, previous research has shown that greater depression severity can hinder engagement with and benefit from digital health interventions [31,43]. Future studies could explore whether additional social factors such as social connectedness and social anxiety act to moderate the relationship between loneliness, suicidal ideation, and depression.

### Strengths and Limitations

The study has several strengths. To our knowledge, this study is the first to examine whether loneliness—a known risk factor for suicidal ideation and depression—affects how young people engage with a digital health intervention. Accordingly, this study contributes new knowledge about the person-specific characteristics that may need to be considered when designing and delivering such interventions. The data for this study was derived from a well-designed randomized controlled trial of a therapeutic smartphone intervention involving 455 young adults and thus provided a large robust sample that supports the reliability of the current findings and conclusions.

There are some limitations to consider. As patient information was collected independently via a web-based survey, self-reporting bias could affect the findings via over- or underestimated effects. The TILS was used because it is a brief validated measure that can reduce survey length and minimize attrition; however, the limited number of items and the small score range (3-9) may not appropriately capture nuance or heterogeneity in loneliness. Future studies focusing primarily on loneliness should consider using more comprehensive scales. Given that loneliness was not a primary or major secondary outcome of the main trial examining the efficacy of LifeBuoy, measures that differentiate between social and emotional loneliness, such as the DeJong-Gierveld Loneliness Scale [45], were not considered and would have allowed for more nuanced analyses of the role of loneliness in digital health interventions. Information on current engagement with mental health treatment was not collected, although whether participants ever received mental health treatment was assessed, and app engagement did not differ on this variable in both intervention and control conditions. Future studies similar to this study should assess both current and past mental health treatment of participants and determine the potential impact of these variables on app engagement. Broader limitations relating to the primary randomized controlled trial have been discussed elsewhere [25].

### Conclusion

Loneliness did not affect how young people engaged with or benefited from a digital health intervention (LifeBuoy) shown to reduce suicidal ideation in young adults. Despite a significant positive association between loneliness and depression levels, loneliness did not affect young people’s engagement with LifeBuoy nor moderate clinical outcomes. As the first study to explore how loneliness may affect young people’s engagement with and benefits from a targeted digital health intervention, this study has developed new insights into whom these self-guided interventions may work best for. Future studies should analyze the interplay of additional factors such as motivation, social connectedness, and social anxiety, as well as distinguish between social and emotional loneliness using more refined measures.

## Appendix

Multimedia Appendix 1 Recruitment advertisement.

Multimedia Appendix 2 CONSORT-eHEALTH checklist (V 1.6.1).

## Abbreviations

BDI: Black Dog Institute
CONSORT: Consolidated Standards of Reporting Trials
PHQ-9: Patient Health Questionnaire
SIDAS: Suicidal Ideation Attributes Scale
TILS: Three-Item Loneliness Scale

## Data Availability

The data sets generated or analyzed during this study are not publicly available due to the sensitive nature of the data but are available from the corresponding author upon reasonable request.
