# Supplementary material for: The Effectiveness of a Smartphone Intervention Targeting Suicidal Ideation in Young Adults: Randomized Controlled Trial Examining the Influence of Loneliness
Source: JMIR Ment Health. 2023 Mar 30;10:e44862. doi: 10.2196/44862 (PMC10131597; doi:10.2196/44862)
Supplement: Multimedia Appendix 1 [file mental_v10i1e44862_app1.docx]

Appendix A

**Recruitment advertisement: Facebook**


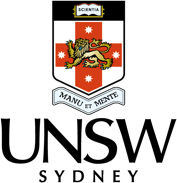

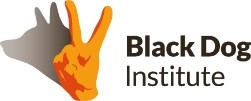


The Black Dog Institute is inviting **young people aged between 18-25 who have experienced suicidal thoughts** to participate in a study evaluating the effectiveness of a smartphone app designed to reduce suicidal thoughts. You will receive **$40** to participate. This study includes 7 modules of the app to be completed over 6 weeks (1-hour total), online surveys completed before (35 minutes) and after using the app (25 minutes), then 3 months after using the app (25 minutes). Click here <Insert the link to the recruitment page> to get more information. Lifeline: 13 11 14.

**Short version:**

The Black Dog Institute is inviting young people **aged between 18-25 who have experienced suicidal thoughts** to evaluate the effectiveness of a smartphone app designed to reduce suicidal thoughts. You will receive $40 for participating. Click here

<Insert the link to the recruitment page> to know more. Lifeline: 13 11 14
